# TESTING A RECRUITMENT STRATEGY FOR A NATIONAL SAMPLE OF OLDER LGBT ADULTS

**DOI:** 10.1093/geroni/igae098.0109

**Published:** 2024-12-31

**Authors:** Phil Schumm, Colm O’Muircheartaigh

**Affiliations:** University of Chicago, Chicago, Illinois, United States; University of Chicago, Chicago, Illinois, United States

## Abstract

This presentation describes a recently completed feasibility study (SAMLAP) of procedures to screen and recruit a nationally-representative, probability sample of older LGBT adults, intended to serve as the basis for a large-scale, longitudinal study of their health trajectories as they age. SAMLAP used push-to-web with an ABS sample of 70,000 addresses in order (i) to identify LGBT household members aged 40 or more, and (ii) to follow up in-person with a sample of about 300 identified individuals. The sample was stratified by region, urbanicity, and legislative environment. We conducted several randomized experiments to test the effectiveness of different recruitment strategies, including: 1) varying incentive amounts and timings, 2) including an SAQ with the first web-push mailing, and 3) implementing a last-ditch contact via FedEx. We also varied invitation wording to test the effectiveness of different approaches to increase participation by sexual and gender minorities (SGM). We discuss the effectiveness of these strategies and suggest best practices to recruit a high-quality sample of older LGBT adults. The overall response rate to the screener was 15%. 13% of screened individuals who were over 40 years of age identified as LGBT; TK3% of households containing an age-eligible LBGT individual contained 2 or more eligible people. Of these eligible individuals, 47% were successfully interviewed either in person or by phone on a range of health and related social factors. We conclude that it is possible to identify a high-quality, U.S. national probability sample of older LGBT adults and to recruit them for follow-up interviews.

